# Small Genome Size Ensures Adaptive Flexibility for an Alpine Ginger

**DOI:** 10.1093/gbe/evaf151

**Published:** 2025-07-24

**Authors:** Qing-Song Xiao, Tomáš Fér, Wen Guo, Hong-Fan Chen, Li Li, Jian-Li Zhao

**Affiliations:** State Key Laboratory of Vegetation Structure, Function and Construction, Ministry of Education Key Laboratory for Transboundary Ecosecurity of Southwest China, School of Ecology and Environmental Science, Yunnan Key Laboratory of Plant Reproductive Adaptation and Evolutionary Ecology, Institute of Biodiversity, Yunnan University, Kunming 650500, China; Department of Botany, Faculty of Science, Charles University, Prague, Czech Republic; State Key Laboratory of Vegetation Structure, Function and Construction, Ministry of Education Key Laboratory for Transboundary Ecosecurity of Southwest China, School of Ecology and Environmental Science, Yunnan Key Laboratory of Plant Reproductive Adaptation and Evolutionary Ecology, Institute of Biodiversity, Yunnan University, Kunming 650500, China; State Key Laboratory of Vegetation Structure, Function and Construction, Ministry of Education Key Laboratory for Transboundary Ecosecurity of Southwest China, School of Ecology and Environmental Science, Yunnan Key Laboratory of Plant Reproductive Adaptation and Evolutionary Ecology, Institute of Biodiversity, Yunnan University, Kunming 650500, China; State Key Laboratory of Vegetation Structure, Function and Construction, Ministry of Education Key Laboratory for Transboundary Ecosecurity of Southwest China, School of Ecology and Environmental Science, Yunnan Key Laboratory of Plant Reproductive Adaptation and Evolutionary Ecology, Institute of Biodiversity, Yunnan University, Kunming 650500, China; State Key Laboratory of Vegetation Structure, Function and Construction, Ministry of Education Key Laboratory for Transboundary Ecosecurity of Southwest China, School of Ecology and Environmental Science, Yunnan Key Laboratory of Plant Reproductive Adaptation and Evolutionary Ecology, Institute of Biodiversity, Yunnan University, Kunming 650500, China

**Keywords:** intraspecific genome size, adaptation, stomatal traits, common garden, environmental factors, *Roscoea tibetica*

## Abstract

Understanding the proximate and ultimate causes of genome size variation has been the focus of considerable research. However, the extent and cause of intraspecific variation in genome size are debated and poorly understood. This study aimed to test the role of genome size in adaptation through variations in intraspecific genome size. Genome size was measured in 53 *Roscoea tibetica* populations from the Hengduan Mountains using flow cytometry. Stomatal size and density data were collected from wild and common garden populations. Associations among genome size, environmental factors, and stomatal traits were explored. We found that high genome size variability was positively correlated with most environmental factors but negatively correlated with solar radiation during the growing season. The environment, rather than geography, significantly influenced variations in genome size. Stomatal traits measured in the wild were significantly correlated with genome size, but no such correlations were detected in the common garden. Populations in the common garden had larger stomatal sizes and lower stomatal densities. Populations with smaller genome size presented a larger degree of stomatal trait variation from the wild to the common garden. Our findings suggest that intraspecific genome size has undergone adaptive evolution driven by environmental stress. A smaller genome size is more advantageous for the alpine ginger to adapt to and thrive in changing alpine habitats.

Significance Although genome size (GS) variation has been widely studied, the mechanisms through which intraspecific GS variation occurs across diverse habitats remain poorly understood. We examined variations in the intraspecific GS of the alpine herb *Roscoea tibetica*. Our study emphasizes the complex interplay between environmental factors and GS variation. A smaller GS is more advantageous to the alpine ginger, and stomatal traits do not reliably predict intraspecific GS variation due to plasticity.

## Introduction

Variations in genome size (GS) play a crucial role in evolution and ecological adaptation (Bennett 1987; Beaulieu et al. 2008; Yang et al. 2013; Schubert and Vu, 2016; Marais et al. 2020; Bureš et al. 2024). Although numerous studies have explored the reasons for GS variation, the substantial variation in GS among living beings remains a fascinating but puzzling question (Dkennedy and Norman 2005; Sanders et al. 2021; Armstrong et al. 2023).

Several hypotheses have been proposed from different perspectives regarding the variation of GS and the origin of genome complexity (Bennett 1977; Bureš et al. 2024; Cavalier-Smith 1978, 2005; Gregory and Hebert 1999; Hessen et al. 2010; Knight et al. 2005). The genome-streamlining (Hessen et al. 2010) hypothesis proposes that metabolic resources, such as nitrogen (N) and phosphorus (P), play an important role in GS selection. As N and P are the main components of DNA, individuals with larger genomes are at a disadvantage when N and P are limited (Acquisti et al. 2009; Hessen et al. 2010; Leitch et al. 2014; Guignard et al. 2016; Faizullah et al. 2021). However, when N and P are sufficient, grassland plants with larger genomes accumulate more aboveground biomass and are more competitive than those with smaller genomes (Peng et al. 2022). The large-genome-constraint hypothesis suggests that a larger GS produces a larger cell volume, which limits physiological activity (Knight et al. 2005; Veselý et al. 2020; Théroux-Rancourt et al. 2021; Šmarda et al. 2023), decreases the cell division rate (Šímová and Herben 2012), and increases plant N and P requirements (Peng et al. 2022). One likely hypothesis is that GS determines the minimum cell size rather than cell size in general (Bennett 1987; Šímová and Herben 2012; Roddy et al. 2020). The genome-streamlining hypothesis is not equivalent to the large-genome-constraint hypothesis. The latter offers a broader perspective, implying that large genomes restrict the plasticity of species or individuals in GS-related traits, thereby narrowing the range of environmental conditions in which a species or individual can thrive. However, the term “constraint” may not be accurate because it can be a misleading placeholder for “natural selection” (Olson 2019). Thus, we use the term large-genome hypothesis instead of the large-genome-constraint hypothesis hereafter. Nevertheless, we can propose that the contrasting extent of plasticity among species or individuals may be related to different GS. Thus, a larger GS may be disadvantageous for species in harsh environments owing to a lower extent of plasticity.

However, evidence for the large-genome hypothesis comes primarily from between-species comparisons and phenotypic variation in the wild (Knight et al. 2005; Šmarda et al. 2010; Roddy et al. 2020; Veselý et al. 2020; Théroux-Rancourt et al. 2021; Peng et al. 2022; Šmarda et al. 2023; Dastpak et al. 2025). However, whether this hypothesis applies to intraspecific GS variability remains largely unknown. Additionally, the correlation between GS variation and phenotypic characteristics from the wild to the common garden has not yet been elucidated. The GS of individuals should be identical, regardless of whether they are growing in the wild or in a common garden. Assuming that the large-genome hypothesis of GS evolution is universal, the correlation between intraspecific GS and phenotypic variation (plasticity), such as cell size, should also not be constant between populations in the wild and common gardens. To date, this assumption has not been tested within species by comparing the phenotypic characteristics of wild and common gardens.

**Fig. 1.**
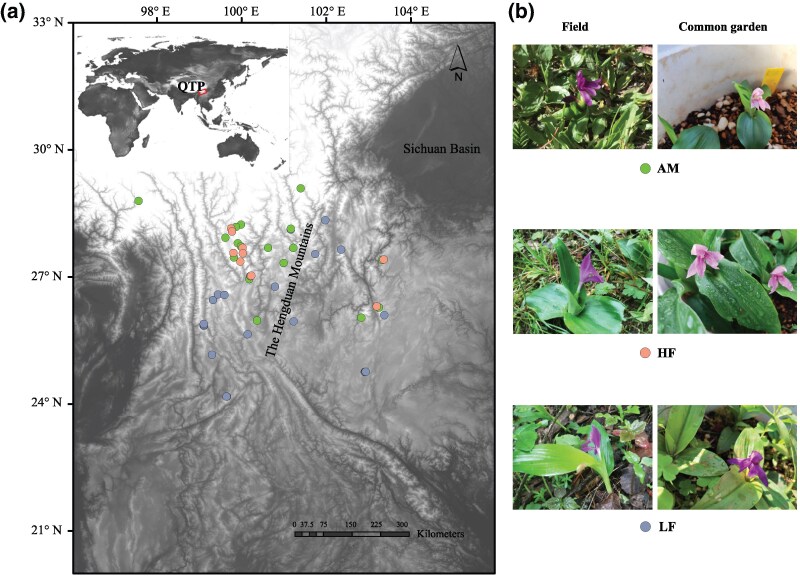
Sampling sites across the entire distribution range and intraspecific GS variation of *R. tibetica*. a) Map of the collection locations of *R. tibetica*. The panorama at the upper left corner illustrates the distribution of *R. tibetica* in the southeast of QTP. b) Morphological diversity in *R. tibetica* from field (the upper row) and common garden (the lower row). AM, alpine meadow type; HF, high-altitude forest type; LF, low-altitude forest type; QTP, Qinghai-Tibetan Plateau.

*Roscoea tibetica* (Zingiberaceae), an alpine ginger in *Roscoea*, is widely distributed in the biodiversity hotspot area of the Hengduan Mountains, from low-altitude forests (LFs) to high-altitude meadows, with large morphological variation (Fig. 1 a and b) (Li et al. 2021). A previous study revealed that *R. tibetica* has three ecotypes based on morphological and evolutionary differences: alpine meadow (AM), high-altitude forest (HF), and LF (Li et al. 2023). The names of the three ecotypes were coined according to their habitats. These three ecotypes exhibited significant differences in flower morphology, number of leaves, and plant height. In terms of evolutionary relationships, thousands of unlinked single-nucleotide polymorphisms strongly indicate that these three ecotypes are reciprocally monophyletic lineages (Li et al. 2023). The large morphological and ecological variations in *R. tibetica* provide an opportunity to test the applicability of the large-genome hypothesis to intraspecific GS variation in plasticity. Here, according to the large-genome hypothesis for *R. tibetica*, we assumed that (i) the harsher environment at higher elevations has a smaller GS in the wild populations and (ii) the smaller GS exhibits larger variation in morphological traits (plasticity), such as stomatal size and density, between wild and common garden populations. To test these hypotheses, the correlations between GS and environmental factors, as well as between GS and morphological traits under wild and common garden conditions, were evaluated. Based on these correlations, we aimed to determine whether the large-genome hypothesis could sufficiently explain the geographical patterns of intraspecific GS variation.

## Results

### Estimation of GS

**Fig. 2.**
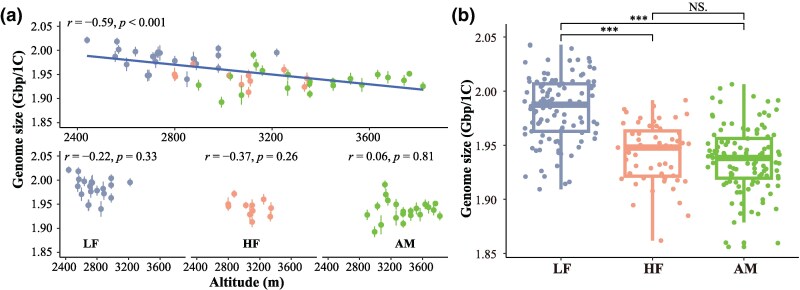
GS variation of wild *R. tibetica* populations with altitude. a) Correlations between entire GS and altitude (above) and between GS within each ecotype and altitude (below). *r* is the correlation coefficient, and *P* indicates the significant level of *r*. The whiskers represent the standard errors. b) Differences in GS among the three ecotypes. AM, alpine meadow type; HF, high-altitude forest type; LF, low-altitude forest type. Significance levels are denoted by asterisks: ***P < 0.001; NS = nonsignificant.

This study measured GS based on the 1C value in 53 populations and included 265 individuals of *R. tibetica* (Tables S1 and S2). The mean GS was approximately 1,956.76 Mb, ranging from 1,892.59 to 2,018.32 Mb with approximately 1.066-fold variation (Table S1). The GS values for all three ecotypes decreased linearly with increasing altitude (Fig. 2a). The mean GS of the LF ecotype was largest (1,984.00 ± 30.39 Mb), followed by that of HF (1,942.40 ± 27.89 Mb), and AM had the smallest value (1,937.40 ± 30.50 Mb) (Fig. 2). Moreover, the GS of the LF group was significantly higher than those of the HF and AM groups (*P* < 0.001; Fig. 2b). The GS showed no significant difference between the two high-elevation ecotypes, HF and AM.

### Correlation Between GS and Environmental Factors

**Fig. 3.**
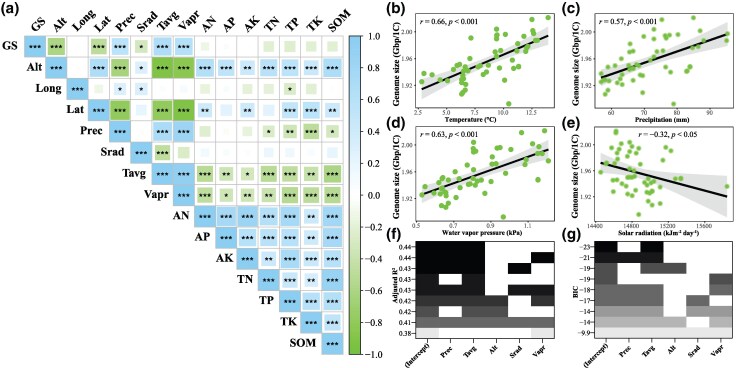
Correlations between GS and environmental factors for *R. tibetica*. a) Pearson's correlation coefficients among GS and geographic factors (altitude, longitude, and latitude), climatic factors (Prec, Srad, Tavg, and Vapr), and soil nutrients (AN, AP, AK, TN, TP, TK, and SOM). Srad is the solar radiation of the growing season from June to August. Significance levels are denoted by asterisks: *P < 0.05; **P < 0.01; and ***P < 0.001. Alt, altitude; Long, longitude; Lat, latitude; Prec, monthly mean precipitation; Srad, solar radiation of growth season from June to August; Tavg, monthly mean temperature; Vapr, water vapor pressure; AN, alkali-hydrosoluble nitrogen; AP, available phosphorus; AK, available potassium; TN, total nitrogen; TP, total phosphorus; TK, total potassium; SOM, soil organic matter. b to e) Scatter plots showing the relationship between GS and four climatic factors. The black regression lines represent the least-squares estimates of the conditional mean function, and the 95% confidence interval is shown by the grey shading around the line. Model selection through adjusted *R*^2^ estimated by regression f) and Schwarz's information criterion (BIC) g).

Overall, GS showed a significant positive correlation with monthly climatic factors (Fig. S1). GS was positively correlated with monthly mean temperature (*r* = 0.66, *P* < 0.001), monthly mean precipitation (*r* = 0.57, *P* < 0.001), and water vapor pressure (*r* = 0.63, *P* < 0.001; Fig. 3a to d). However, solar radiation in June, July, and August was negatively correlated with GS, and only in July, the correlation was significant (*r* = −0.42, *P* < 0.01; Fig. S2). Since the growing season for *R. tibetica* consists of June, July, and August, the growing season solar radiation included only these three months in the following analysis. The results indicated that GS was significantly negatively correlated with solar radiation during the growing season (*r* = −0.32, *P* < 0.05; Fig. 3a and e). Our results showed a significant negative correlation between GS and altitude (*r* = −0.59, *P* < 0.001; Fig. 2a). Within ecotypes, the correlation between GS and altitude was not significant. In addition, a significant negative correlation was found between the GS of *R. tibetica* and latitude (*r* = −0.49, *P* < 0.001; Fig. 3a). GS showed no correlation with longitude or soil nutrients (Fig. 3a). In addition, with increasing altitude, precipitation, temperature, and water vapor pressure decreased, whereas solar radiation and soil nutrients increased (Fig. 3a). For stomatal traits from the wild and common garden conditions, no significant correlations were found between stomatal size and density on either the abaxial or adaxial sides of the leaves and environmental factors, except for significant positive correlations between stomatal size and precipitation in the wild populations (*P* < 0.01; Fig. S3). Moreover, significant negative correlations between stomatal size and density were observed either on the abaxial or adaxial sides of leaves for the wild populations (*P* < 0.001), whereas such significant correlations were not observed for the traits from common garden populations (*P* > 0.05; Fig. 5; Fig. S3).

**Fig. 4.**
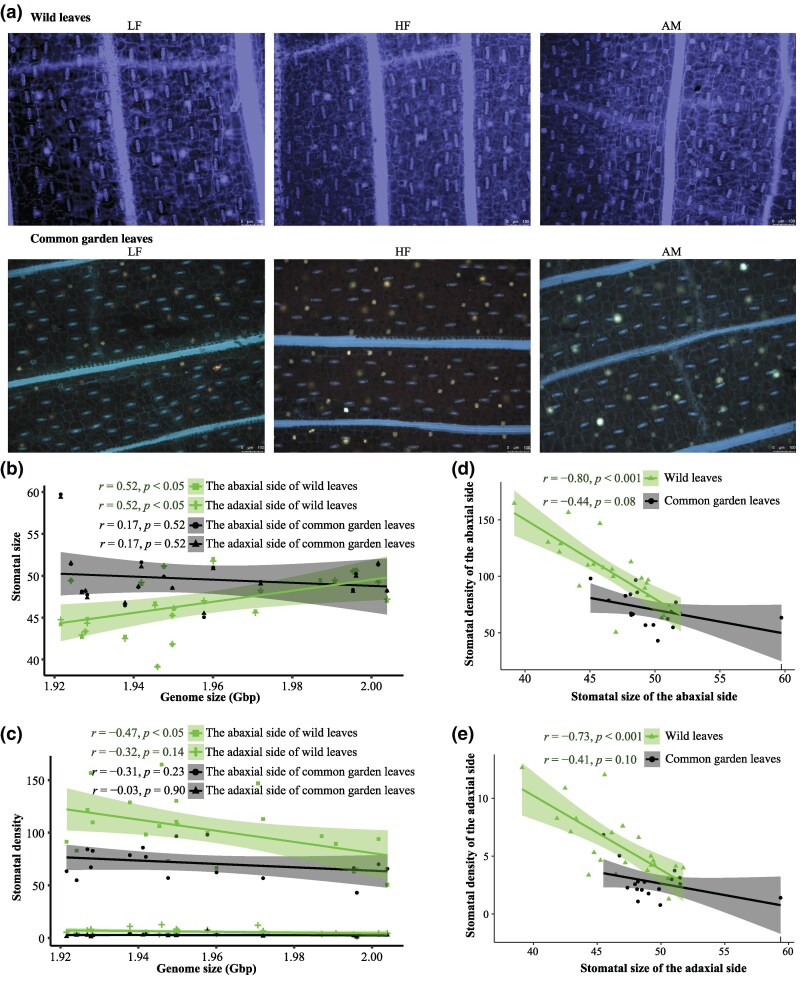
Plasticity of stomatal size and density from wild to common garden. a) Photographs of stomatal traits of different ecotypes of *R. tibetica* from wild and common garden leaves. LF, low-altitude forest type; HF, high-altitude forest type; AM, alpine meadow type. b, c) Correlations between GS and stomatal size and stomatal density, respectively. d, e) Correlations between stomatal size and density of the abaxial and adaxial of leaves. The regression lines represent the least-squares estimate of the conditional mean function, and 95% confidence interval is shown by the shading around the line. Stomatal size and stomatal density of the abaxial and adaxial surfaces of leaves were measured.

**Fig. 5.**
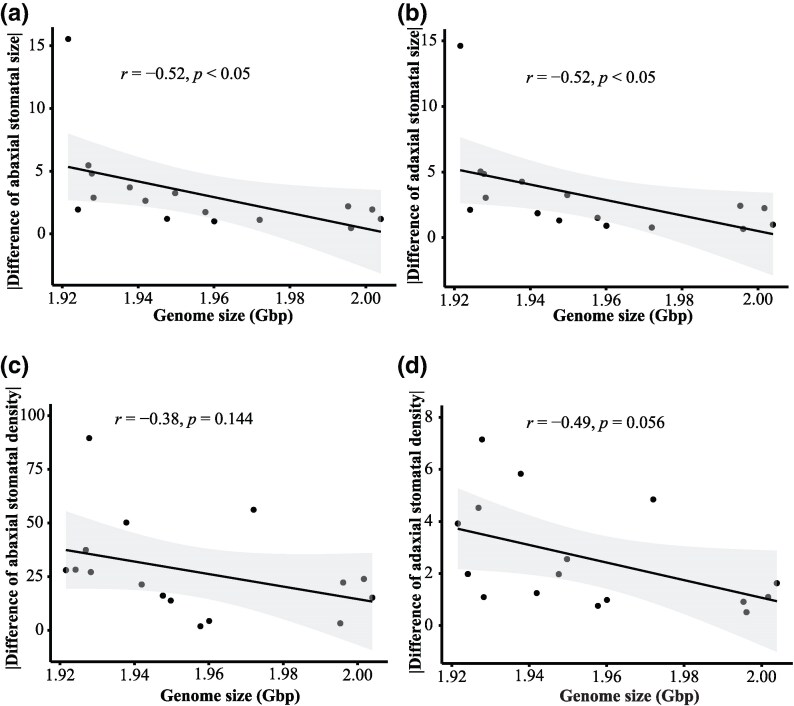
Correlations between GS and absolute stomatal traits differences (abs(Trait[wild] − Trait[common garden])). Double vertical lines represent absolute values.

Regression subset analysis indicated that precipitation + temperature and precipitation + temperature + water vapor pressure were two equally good models (both had adjusted *R*^2^ = 0.44; Fig. 3f) that best explained the GS variation. The lowest Bayesian information criterion (BIC) value further indicated that temperature was the best independent variable to explain GS variability (multiple *R*^2^ = 0.44, adjusted *R*^2^ = 0.43, *df* = 51, *P* < 0.001; Fig. 3g). Relative weight analysis suggested that precipitation (*R*^2^ = 0.25) and temperature (*R*^2^ = 0.24) contributed the most to GS variation, followed by water vapor pressure (*R*^2^ = 0.21). The total *R*^2^ value for all the independent variables was 0.47. These analyses suggest that precipitation and temperature can explain GS variation well.

### Contribution of Geography and Environmental Factors to GS Variation

Mantel tests showed a strong positive correlation between GS and both geographic distance (Mantel statistic, *r* = 0.178, *P* < 0.01) and environmental factors (*r* = 0.290, *P* < 0.001) (Table 1). In the partial Mantel tests, when the environment was controlled for, GS exhibited no significant association with geographic distance (*r* = −0.004, *P* > 0.05), whereas it remained positively correlated with the environmental factors when geographic distance was controlled for (*r* = 0.233, *P* < 0.001) (Table 1).

**Table 1:** The relationships between GS variation and geography or environment

Models	Geography		Environments	
	*r*	*P*	*r*	*P*
**Mantel tests**				
Genome size	**0.178**	**<0.01**	**0.290**	**<0.001**
**Partial Mantel tests**				
Environments were controlled	−0.004	0.513	…	…
Geography was controlled	…	…	**0.233**	**<0.001**

*r* is correlation coefficient and *P* is significance level.

### Analysis of the Relationship Between Morphological Traits and GS

The stomatal size and density of *R. tibetica* differed between growth conditions (Fig. 4a). For *R. tibetica* collected from the wild, stomatal size on both the abaxial and adaxial sides of the leaves was positively correlated with GS (*r* = 0.52, *P* < 0.05, and *r* = 0.52, *P* < 0.05, respectively) (Fig. 4b). Stomatal density at the abaxial side of leaves was negatively correlated with GS (*r* = −0.47, *P* < 0.05), while the correlation between stomatal density at the adaxial side and GS was not significant (*r* = −0.32, *P* > 0.05) (Fig. 4c). For plants cultivated in the common garden, no significant correlations were detected between GS and stomatal size and density on both sides of the leaves (Fig. 4b and c).

Stomatal size from wild populations was significantly less than that from common garden populations (abaxial side: *t* = −2.25, *P* < 0.05; adaxial side: *t* = −2.24, *P* < 0.05). Stomatal density in the wild populations was significantly higher than in the common garden populations (abaxial side: *t* = 3.98, *P* < 0.01; adaxial side: *t* = 5.01, *P* < 0.001). More importantly, the absolute values of differences in stomatal size for both sides were negatively and significantly correlated with GS (both *r* = −0.52, *P* < 0.05; Fig. 5a and b). Although the correlations between absolute differences in stomatal density and GS were not significant, these relationships were negative (abaxial side: *r* = −0.38, *P* = 0.144; adaxial side: *r* = −0.49, *P* = 0.056; Fig. 5c and d).

## Discussion

### Smaller GS Promotes *R. tibetica* to Adapt to Higher Elevation

Although the effects of environmental stress on GS evolution have been extensively studied, the role of environmental factors in intraspecific GS variation remains poorly understood (Blommaert 2020; Faizullah et al. 2021). Studies have revealed that the distribution of angiosperm GS is shaped by climate (Simonin and Roddy 2018; Bureš et al. 2024), suggesting that climatic factors play a crucial role in the evolution of GS. Our analysis suggests that variations in intraspecific GS in *R. tibetica* across the Hengduan Mountains are determined by environmental factors rather than being randomly distributed. Previous research has suggested that environmental pressures are the direct driving forces of natural selection and evolutionary change (Hoffmann and Hercus 2000). If the evolution of GS is neutral in *R. tibetica*, no significant association should exist between the GS and environmental factors. The intraspecific GS of *R. tibetica* was significantly related to altitude, latitude, monthly precipitation, monthly average temperature, water vapor, and solar radiation (Figs. 2 and 3). The Mantel test indicated that the environment shapes the geographical pattern of GS variation in *R. tibetica* (Table 1). Among environmental factors, precipitation and temperature were the best predictors of GS variation (Fig. 3f and g), suggesting their crucial roles in the evolution of intraspecific GS. Thus, the close relationship between intraspecific GS and environmental factors suggests that the variations observed across the distribution range of *R. tibetica* are the result of adaptive evolution. This observation mirrors a similar pattern observed in maize, which likely reflects the effects of natural selection on GS variation (Diez et al. 2013; Bilinski et al. 2018).

Environmental stress conditions, such as low CO_2_, water, and nutrient levels, may favor species with smaller genomes (Faizullah et al. 2021). Plants with small genomes are more adaptable to harsh environments (Leitch and Leitch 2013), can maximize water use efficiency and CO_2_ absorption, and exhibit rapid growth in dry or variable water environments (Hetherington and Woodward 2003; Simonin and Roddy 2018; Záveská et al. 2024). We found that, for *R. tibetica*, GS significantly decreased with increasing altitude and solar radiation during the growing season and increased with greater precipitation, temperature, and water vapor pressure (Fig. 2; Fig. 3). Thus, higher altitude, lower precipitation, temperature, water vapor, and stronger solar radiation likely created environmental stress conditions that influenced the evolution of GS in *R. tibetica*. These conditions enable plants with a small GS to survive in harsher environments with increasing elevation.

More importantly, the results supported our assumption based on the large-genome hypothesis in that the smaller GS was associated with a larger variation in stomatal traits between populations from the wild and common gardens (Fig. 5a to d). The large-genome hypothesis emphasized the effects of GS on minimum cell size (Bennett 1987; Šímová and Herben 2012; Roddy et al. 2020; Veselý et al. 2020; Théroux-Rancourt et al. 2021; Bhadra et al. 2023; Šmarda et al. 2023). This hypothesis suggests that species or individuals with a smaller GS have greater plasticity in GS-dependent traits than those with larger genomes. Stomatal size and density are two main traits correlated with GS variation (Beaulieu et al. 2008; Lomax et al. 2014; Simonin and Roddy 2018; Roddy et al. 2020; Veselý et al. 2020; Faizullah et al. 2021). The common garden in our study provided different living conditions for individuals of *R. tibetica* compared to the wild conditions, especially for higher-elevation individuals. *Roscoea tibetica* had a larger stomatal size and lower stomatal density in the common garden than in the wild (Figs. 4 and 5). In such a situation, the degree of variation in the two traits between the wild and common garden populations was negatively correlated with GS (Fig. 4b to e), indicating that populations with a smaller GS from higher elevations exhibited higher plasticity with larger variations in stomatal size and density. This suggests that populations with smaller GS may be more flexible in responding to habitat changes through large-scale changes in morphological traits, which may be an adaptive feature for *R. tibetica* to thrive at higher elevations.

Moreover, several studies have proposed that the genome-streamlining hypothesis holds true in environments with extremely limited amounts of P and N (Kang et al. 2015; Bales and Hersch-Green 2019; Faizullah et al. 2021). However, our study found no correlation between GS and soil nutrients, which does not support the genome-streamlining hypothesis. For *R. tibetica*, soil nutrients increased with altitude (Fig. 3a). This finding suggests that the soil nutrients in the habitats were sufficient and did not create stress conditions, thereby exerting a weak or no effect on GS changes in *R. tibetica*. A potential causal explanation could be that rhizomes, which function as storage organs in Zingiberaceae, prevent plants from being affected by nutrient deficiency (Záveská et al. 2024).

### Stomatal Traits Do Not Reliably Predict Intraspecific GS Variations

Many studies indicate that GS is significantly positively correlated with stomatal size and negatively correlated with stomatal density (Beaulieu et al. 2008; Lomax et al. 2014; Simonin and Roddy 2018; Roddy et al. 2020; Veselý et al. 2020; Faizullah et al. 2021). Current models suggest that selection for the GS is influenced by stomatal size (Faizullah et al. 2021), implying that environmental selection based on stomatal size may influence GS evolution in plants (Veselý et al. 2020). However, several studies have suggested that a reduced GS facilitates increased variability in both stomatal size and density (Roddy et al. 2020; Théroux-Rancourt et al. 2021; Jiang et al. 2023; Dastpak et al. 2025), suggesting that stomatal traits have ample room for plasticity because of resource availability and environmental conditions (Simonin and Roddy 2018). Our observations in the wild clearly indicate that GS is positively correlated with stomatal size and negatively correlated with stomatal density (Fig. 4b and c). However, these significant correlations disappeared when plants from the common garden were analyzed, strongly suggesting the plasticity of stomatal traits. The plasticity of stomatal traits has been reported in other plants (Sun et al. 2014; Wang et al. 2019; Roddy et al. 2020; Théroux-Rancourt et al. 2021; Jiang et al. 2023; Dastpak et al. 2025), suggesting that stomatal traits are controlled by environmental factors rather than by the GS. Moreover, correlations between stomatal size and density changed from the wild to the common garden (Fig. 5; Fig. S3), further suggesting the predominant impact of environmental factors on the plasticity of stomatal traits. A growth chamber experiment on highland teosintes revealed weak support for a positive correlation between GS and cell size (Bilinski et al. 2018), suggesting that the size relationship could be affected by habitat. Comprehensive evolutionary analysis of GS and stomatal size in Proteaceae revealed that ancient changes in GS clearly affected stomatal size in Proteaceae, but adaptive responses to habitat strongly changed the genome–stomatal size relationship, suggesting that environmental adaptation to stomatal size is independent of the effects of GS (Jordan et al. 2015). Thus, Jordan et al. (2015) proposed that a general genome–stomatal size relationship cannot be expected through geological time because habitats and other factors, such as atmospheric CO_2_ concentration, could influence stomatal size independently of GS. Previous studies, along with our findings on stomatal trait differences between wild and common gardens, strongly suggest that general stomatal size may not reliably predict intraspecific GS variations, while minimum cell size may (Jiang et al. 2023).

Both GS and stomatal size are affected by environmental factors. Thus, a causal relationship between stomatal traits in the wild and the GS is likely to be dominated by environmental factors. Moreover, the decoupling of the genome–stomatal size relationship observed when analyzing traits from common gardens suggests that selection pressures are likely to be reduced in controlled environments such as common gardens.

## Conclusions

This study elucidates the adaptive evolution of intraspecific GS variation in the alpine herb *R. tibetica* from two perspectives. First, environmental factors, especially precipitation and temperature, are the key drivers that allow plants with large GSs to survive in high-altitude environments. Second, stomatal traits do not reliably predict intraspecific GS variations observed in wild and common garden experiments, suggesting that the genome–stomatal size relationship can change. In general, these results illustrate the applicability of the large-genome hypothesis to the adaptive evolution of intraspecific GS. Importantly, this study suggests that a small GS is beneficial for alpine plants living in harsh environments. Although our inferences were from only one species, our findings advance the understanding of intraspecific GS evolution. These findings also suggest that *R. tibetica* serves as a valuable model for further exploration of genomic signals for intraspecific GS variation and how GS promotes adaptive evolution in heterogeneous mountainous environments.

## Materials and Methods

### Sampling

We conducted a comprehensive sampling of *R. tibetica* across an altitudinal range of 2,400 to 3,800 m. To examine variations in morphological traits, such as stomatal size and density, we collected samples from a common garden. For each population, approximately 20 individual living plants with rhizomes were collected for cultivation in a common garden at Yunnan University, Kunming, China (102.8548° E, 24.8291° N). Young, fresh leaves were harvested from 53 common garden populations for GS measurements 1 year after replanting, including 21 populations of LF, 11 populations of HF, and 21 populations of AM (Fig. 1a; Tables S1 and S2). Leaves of five individuals were randomly selected from each population for GS measurements, and the GS of 265 individuals was measured.

To measure stomatal traits, owing to the difficulty in collecting mature leaves from all 53 populations during the flowering stage within a single year, we endeavored to obtain mature leaves from 23 wild populations in 1 year and 17 common garden populations in the following year (Table S3) to mitigate the impact of annual fluctuations in morphological traits on the results. Approximately 6 to 10 leaves were collected from each population sampled. A total of 218 leaves were collected from the field. The leaves were dried using silica gel, flattened in a small penetrative bag, and transported to the laboratory. A total of 150 leaves were collected from common gardens and dried using silica gel.

### GS Measurements

The nuclear DNA content (1C value) of *R. tibetica* was measured by flow cytometry. Using the internal standard method, the nuclei of the samples were stained using a two-step method with propidium iodide (PI) fluorescent dye (Greilhuber et al. 2007; Pellicer et al. 2021). We mixed 50 mg of leaves of *R. tibetica* and 50 mg of internal standard tomato (1C = 0.958 Gb) with 500-µL nuclei extraction buffer in a plastic Petri dish. This standard tomato, planted by us, was calibrated by a tomato (∼0.9 Gb) from the Kunming Institute of Botany of the Chinese Academy of Sciences and by maize (∼2.2 Gb) from the research of Li et al. (2019). Leaf tissues were chopped to dissociate the cells into a buffer (CyStain, Sysmex Partc GmbH, Czech Republic) and incubated for 30 to 90 s at room temperature (∼25 °C). Cell solutions were filtered through a 50-µm CellTris filter into centrifugal tubes with 2-mL PI staining solution (CyStain) and incubated for 15 to 30 min in the dark at room temperature. Finally, the prepared sample solutions were analyzed using a flow cytometer (CyFlow Space; Sysmex Europe SE, Norderstedt, Germany). The minimum number of nuclei for the sample to be tested was set to 5,000 nuclei, and the coefficients of variation (CV) of the sample and standard peaks in the flow histogram were always <5%, with a mean of 3.8% (Table S2). The 1C DNA content of samples was calculated using the following equation: nuclear DNA content (Gb) = 0.958 × (mean position of the reference standard peak/mean position of the reference sample peak) (Loureiro et al. 2007). To minimize the artificial variations that may be caused by experimental processes (e.g. Doležel and Bartoš 2005; Noirot et al. 2005; Bennett et al. 2008; Praca-Fontes et al. 2011; Trávníček et al. 2015; Nix et al. 2024), including methodological and plant environmental variables, five individuals were randomly collected from common gardens at the same growth stage. Furthermore, the internal standard was always from the same tomato plant, all samples were measured within 2 h of removal from the plant, all measurements were conducted by one person (Q.-S.X.), only CyStain from a single batch was used for staining, and all estimations were performed using a CyFlow Space flow cytometer. Not all estimations can be completed within a day. These variations may be caused by different conditions, such as room temperature and the degree of chopping. To exclude the effect of artificial variation, we selected ten individuals of *R. tibetica* to estimate GS three times on three different days. The CV was very low at 0.176% to 1.445% (Table S4), indicating the reliability of GS estimation. Based on the results of the GS estimation, this species showed no polyploidy.

### Leaf Trait Measurements

Leaves were trimmed using scissors to measure stomatal size and number. The middle part of the leaf was retained and soaked in double-distilled water for 2 to 3 d. The soaked leaves were placed on glass slides, and the stomata from five different fields of view on the abaxial and adaxial sides of leaves were observed and photographed using a fluorescence microscope (Leica DM5000B). The photographs were imported into ImageJ (Rueden et al. 2017) to measure the size and number of stomata on the abaxial and adaxial sides of the leaves. The stomatal size was represented by the length of the guard cell, which can be estimated by multiplying the guard cell length by a constant factor of 0.36 (de Boer et al. 2016; Jiang et al. 2023). This approach indicated a strong positive correlation between stomatal size and guard cell length. As the stomatal number for all samples was measured in the same visual field and scale, it can also serve as a measure of stomatal density.

### Environmental Data Collection

The climate data, including monthly mean precipitation (Prec, mm), solar radiation (Srad, kJ m^−2^ d^−1^), monthly mean temperature (Tavg, °C), and water vapor pressure (Vapr, kPa) from January to December, were downloaded from the WorldClim version 2.1 with a resolution of 2.5 min (https://www.worldclim.org) (Fick and Hijmans 2017). Soil data with a resolution of 30 arcseconds were downloaded from the Land-Atmosphere Interaction Research Group on the Sun Yat-sen University website (http://globalchange.bnu.edu.cn/research/soil2) (Shangguan et al. 2013). Soil data included alkali-hydrosoluble nitrogen (AN, mg/kg), available phosphorus (AP, mg/kg), available potassium (AK, mg/kg), total nitrogen (TN, g/100 g), total phosphorus (TP, g/100 g), total potassium (TK, g/100 g), and soil organic matter (SOM, g/100 g).

### Statistical Analyses

The significance of differences in GS among the three ecotypes was examined using the *t*-test functions “ggplot” and “geom_signif” in the R packages *ggplot2* (Wickham 2016) and using *ggsignif* to visualize the result of the *t*-test (Ahlmann-Eltze and Patil 2021), respectively, with *P* < 0.05, indicating a significant difference. We performed Pearson's correlation analyses in the *corrplot* (Simko 2021) and *ggplot* packages to estimate the relationships between intraspecific GS and geographical factors (altitude, longitude, and latitude), climatic factors (Prec, Srad, Tavg, and Vapr), and soil nutrients (AN, AP, AK, TN, TP, TK, and SOM). Furthermore, *P* < 0.05, *P* < 0.01, and *P* < 0.001 indicate the significance levels of Pearson's correlations. To explore which climate factors can predict intraspecific GS variation in *R. tibetica*, we performed regression subset selection by exhaustive search using the “regsubsets” function in the *leaps* package (Lumley 2020). This function calculates the adjusted *R*^2^ for different generalized linear models based on different climatic factors. The best model was selected using the adjusted *R*^2^, where the higher the value, the better the model. The BIC was then used to select the optimal and simplest model through all-subset regression analysis using the *leaps* package. The optimal model was indicated by the lowest BIC, and the adjusted *R*^2^ with *P* < 0.05 was used to support the model. To validate the predictions of “regsubsets” and determine the relative contribution of climatic factors to GS, a relative weight analysis was conducted using the “relWeights” function (https://rdrr.io/github/jgodet/utilitR/man/relWeights.html). For both stepwise regression and relative weight analysis, GS served as the dependent variable and climatic factors as the independent variables. All analyses were performed using R version 4.0.3 (R Core Team 2021).

To test the influence of geography and environment on intraspecific GS variation, we performed Mantel and partial Mantel tests with the R package *vegan* (Oksanen et al. 2018) using the “mantel” and “mantel.Partial” functions. To eliminate multicollinearity among factors, we performed principal component analysis on altitude and climate factors using the functions “prcomp” and “ggplot” in the *ggplot2* package and then used the first and second principal component data to calculate the environmental parameter distance between populations. Based on the longitude and latitude information of each population, the geographic Euclidean distance between populations was calculated using the R package *geosphere* (Hijmans 2019) with the function “distm.” For the partial Mantel tests, geography and environment were controlled separately.

We analyzed Pearson's correlations between stomatal traits from wild and common gardens and intraspecific GS using the R packages *ggplot2* and *ggpubr* (Kassambara 2020). The “corrplot” function in the R package *corrplot* was used to test the relationships between morphological traits and environmental factors using Pearson's correlations. Correlations between stomatal size and density were estimated using the same method. These correlations were judged by the correlation coefficients (*r*) and *P*-values, where *P* < 0.05 indicated a significant correlation.

To explore the association between stomatal trait variation and GS, a pairwise *t*-test in the R function *t.test* was used to compare the trait differences for the same wild and common populations. If *t* (mean value of difference) was negative with *P* < 0.05, stomatal traits in wild populations were significantly lower than those in common garden populations. If *t* was positive with *P* < 0.05, stomatal traits in the wild populations are significantly larger. Correlations between the absolute values of the differences in stomatal traits (traits of wild populations minus traits of common garden populations) and GS were estimated using *ggpmisc* and *ggpubr*.

## Supplementary Material

evaf151_Supplementary_Data

## Data Availability

All data for this work are available in Tables S1 to S4.
